# Ethics Requirement Score: new tool for evaluating ethics in publications

**DOI:** 10.1590/S1679-45082014AO3001

**Published:** 2014

**Authors:** Lígia Gabrielle dos Santos, Ana Carolina da Costa e Fonseca, Claudia Giuliano Bica

**Affiliations:** 1 Universidade Federal de Ciências da Saúde de Porto Alegre, Porto Alegre, RS, Brazil.

**Keywords:** Ethics, Research, ethics, Impact factor, Scientific and technical publications

## Abstract

**Objective:**

To analyze ethical standards considered by health-related scientific journals, and to prepare the Ethics Requirement Score, a bibliometric index to be applied to scientific healthcare journals in order to evaluate criteria for ethics in scientific publication.

**Methods:**

Journals related to healthcare selected by the Journal of Citation Reports™ 2010 database were considered as experimental units. Parameters related to publication ethics were analyzed for each journal. These parameters were acquired by analyzing the author’s guidelines or instructions in each journal website. The parameters considered were approval by an Internal Review Board, Declaration of Helsinki or Resolution 196/96, recommendations on plagiarism, need for application of Informed Consent Forms with the volunteers, declaration of confidentiality of patients, record in the database for clinical trials (if applicable), conflict of interest disclosure, and funding sources statement. Each item was analyzed considering their presence or absence.

**Result:**

The foreign journals had a significantly higher Impact Factor than the Brazilian journals, however, no significant results were observed in relation to the Ethics Requirement Score. There was no correlation between the Ethics Requirement Score and the Impact Factor.

**Conclusion:**

Although the Impact Factor of foreigner journals was considerably higher than that of the Brazilian publications, the results showed that the Impact Factor has no correlation with the proposed score. This allows us to state that the ethical requirements for publication in biomedical journals are not related to the comprehensiveness or scope of the journal.

## INTRODUCTION

### Ethics in research

It is undeniable that over the last 100 years, biomedical science has evolved substantially due to the understanding that research is necessary, although, this great advance depends on experimentation.^(1)^ The set of activities that have the objective of developing and contributing towards generalizable knowledge are known as “research.” Generalizable knowledge may be understood as theories, principles, relations, or as the accumulation of information on which it is based, which can be proven by scientific methods.^(2, 3)^ Understanding the need and the importance of experimentation in research, the big question is how to conduct it in an adequate manner, and in a certain sense, ethically.

Along history, important ancient (since the time of Hippocrates), medieval, and modern writings have demonstrated concern with healthcare, although it was only around the mid-20^th^ century that the first international document appeared with norms for medical research with human beings:^(4)^ the Nuremberg Code.^(5)^ This document was approved by various posterior documents, such as the Declaration of Helsinki (1964) in all its versions, the Belmont Report (1978), the International Directives for Biomedical Research in Human Beings (Brazilian translation, 1993) and, in Brazil, the Resolution of the National Council of Health (CNS) 196/96.^(6)^ In 1947, the Nuremberg Code was written by American physicians with the objective of granting subsidies to the Nuremberg Tribunal for judging the so-called crimes committed against humanity in research that was conducted in concentration camps,^(7-10)^ thus becoming the first code of conduct in research that would become internationally accepted.^(11)^

The Nuremberg Code is composed of ten principles that seek to protect the right of subjects that participate in research. The most important aspect of this code is the mandatory nature of the Informed Consent in studies with human beings. Posteriorly, the principles contained in the Nuremberg Code became a part of the codes of ethics of professionals that carry out research, with the purpose of its regulation.^(12)^

Due to the need for regulation of research with human beings, with the intent of protecting populations submitted to it, and due to the small influence the Nuremberg Code had on research practices, the Declaration of Helsinki appeared.^(2)^ The Declaration of Helsinki was published in 1964, during the 18^th ^World Medical Assembly, carried out in Helsinki, and posteriorly revised in 1975 (Tokyo), 1983 (Venice), 1989 (Hong Kong), 1996 (Somerset West), 2000 (Edinburgh), and 2008 (Seoul), and points out the importance of the ethical principles in the performance of research with human beings, establishing aspects such as obtaining the Informed Consent and the prior appreciation of the experimental protocol by a committee independent from the investigator, as per items 15 and 24 (World Medical Association: Declaration of Helsinki, 1964).^(13)^ It also asserts that experiments done outside of the norms of this declaration should not be accepted for publication. Research in animals during this period did not seem to present any ethical problems.

In 1978, the Belmont Report was disclosed, which is considered a theoretic mark for the practice of research and which gave origin to the theory of principles, proposed by Tom Beauchamp and James Childress in the book *Principles of Biomedical Ethics* , published in 1979. The Belmont Report pointed out the following principles as fundamental references for ethics in research:

– Respect for people, that encounters it practical correspondence in the formulation and attainment of the Informed Consent Form;
– Beneficence, which presupposes safety and well-being to the participants for their insightful evaluation of the cost/benefit relation by means of their insertion into the research;
– Justice in the liberal sense of equity and translated by the possibility of equality of access to participation in studies and in the distribution of the results.^(7)^

On October 10^th^, 1996, in Brazil, the CNS Resolution 196/96 was published, creating the Research Ethics Committees (CEPs, acronym in Portuguese) linked to the research institutes and requiring that their composition be multidisciplinary, necessarily including one representative of the community. This resolution is a regulatory mark for Brazilian scientific research involving human experimentation. Such a resolution was prepared with the intention of protecting the participants, the institutions involved, and the State. Also created was the National Commission on Ethics in Research (CONEP), the maximal agent of the area, linked to the CNS – Ministry of Health (MS). It is important to point out how late this process happened in Brazil, since before these agents and regulations, dated 1996, research studies were already conducted. Recently, Resolution 196/96 was revoked by Resolution 466, dated December 12^th^, 2012, and despite presenting significant alterations, there are severe problems in the text. This new version was not used in this study as it was published after data collection.

### Ethics for publication in scientific journals

The concern with the integrity and completeness of scientific reports is ancient. In the fourth methodological principal established by Reneé Descartes, in his *Discourse on the Method* , it is suggested that “in all the parts methodological relations practices should be so complete and reviews so complete that I could be sure that nothing had been omitted.”^(14)^ Based on this principle, one notes the evident importance of consistency and integrity of scientific reports. Obviously, the dynamic of current scientific publications differs from that observed during the 15^th^ century, when in addition to the need for entire reports, other items became important.

Currently, the credibility and prestige of a scientific journal are related to the rigor of the editorial policies, with the publication of studies that were conducted with ethical and scientific rigor, and that have the potential to influence the development of the area of research in which it is inserted.^(15)^

The structural characteristics for the submission of an article vary according to the journal, which currently obliges publishers to establish standardized instructions, trying to make the publication format uniform. The dynamics of science divulged by the journals started to be the object of analysis of publishers who felt the need to structure the publications according to several guidelines, which posteriorly, became recommendations and norms.^(15)^ Within this context, one should emphasize the work carried out by the International Committee of Medical Journal Editors (ICMJE), better known as the Vancouver Group, with the publication of Uniform Requirements for Manuscripts Submitted to Biomedical Journals: Writing and Editing for Biomedical Journals, which as of 1978 seeks to establish guidelines for standardization and characteristics of biomedical journals.^(16)^ However, over time, other concerns appeared which went beyond the preparation of manuscripts. The intention of more recent editions is that of clarifying and directing interests as to rights, privileges, and method description, among other topics.^(17)^

More recently, in 1997, the Committee on Publication Ethics (COPE) was created by a small group of publishers of the United Kingdom. Currently, with more than 7,000 members worldwide in different academic areas, COPE offers counseling for editors and publishers on all the ethical aspects in scientific publications and in particular, on how to deal with cases of poor conduct in research and publication.^(18)^

With the great increase in worldwide scientific production, there is also increased concern in the scientific community with ethical transgressions in scientific publications. Due to the fact that research is linked to values and norms, it is expected that the researcher conduct a study with integrity and that scientific standards of excellence and confidence in its development be guaranteed in its development.^(19)^ Serious violations of this behavior have been known as “poor scientific conduct”, and can be summarized in fabrication, falsification, or plagiarism in preparation of the proposal, in carrying out the study, or in evaluating it, or yet, in reporting the results of the investigation.^(18)^

Due to the great number of clinical trials, the investment of pharmaceutical industries in researching new medications, and the possible ethical problems applicable to these cases (such as omission and non-publication of data), the Consolidated Standards of Reporting Trials (CONSORT) was created by the Consort Group. CONSORT lists and systematizes various initiatives developed to solve problems resulting from the inadequate form data is furnished in randomized clinical trials.^(20)^ In this way, for the transparency of a clinical trial, it should be registered in a database that follows the CONSORT guidelines, such as, for example, the Australian Clinical Trials Registry, the Clinical Trials, the Nederland’s Trial Register, and UMIN Clinical Trials Registry, which are the largest databases in the world. To each randomized clinical study indexed in one of these databases, a registration number is given. This registration confers reliability to the results, since regardless of the outcome of the study, its results are recorded. None of the journals cited the Brazilian registry of clinical trials, ReBEC.

The increased pressure on the investigators to publish at any cost, with the objectives of prestige and more financial aid for research, contributed towards a great part of the growth of fraud in research.^(19)^ One study by the provider of academic data, Thomson Reuters, showed that the number of articles published in journals with peer system evaluation over the last 20 years doubled, while the number of retractions increased 20 times during the same period, possibly as a consequence of the appearance of better plagiarism detection systems such as *Déjà vu* , Ephorus, Jplag, and Etblast, for example,^(21)^ as well as the responsibility that the publishers have received for measures against poor conduct.^(22)^

In Brazil, it is no different. Due to the increase in scientific production, the search for publications is ever increasing, preferentially in journals with a greater Impact Factor (IF).^(23)^ In this sense, various bibliometric indexes have been created to quantify and qualify the scientific production, and are applied to the investigator (considering all his/her articles), to an article independently, or to a publication. Among these, we emphasize the H Index, the Eigenfactor, and the Immediacy Index. Despite the growing importance of these indexes to bibliometry and scientometry worldwide, the most used index is the IF. This index, created by the Institute for Scientific Information (ISI), a part of Thomson Reuters, is applied to each periodic publication and is basically a ratio between citations received and articles published.^(24)^ Explained in greater detail, the IF is calculated by the number of citations (C) that a journal received within a given year, by the number of all the articles it published during the two posterior years (A). In this way, its formula is: IF= C/A (http://thomsonreuters.com/products_services/science/academic/impact_factor/).

Studies that evaluate scientific production enable a panorama of scientific management policies, thus facilitating comprehension of the dissemination of scientific knowledge.^(25)^ Within this context of increasing scientific production worldwide and in face of the cases of fraud in publication, the ethics requirement needs to be valued as a characteristic that adds quality to scientific publications.

This paper proposes to create an Ethics Requirement Score (ERS) with the intent of evaluating the ethics requirement of scientific periodicals in the healthcare area. More important that publishing is the concern with respect and with the integrity in which the research is conducted. The ERS should show if there is an association between the IF of the journal and the ethical requirements in the instructions given to the authors.

The relevance of this study was in verifying if the ethical aspects that seek the benefits and rights of the study subjects are respected, and if the interests of the sponsors, researchers, and investigators are duly reported, among other aspects. Because of the consultation of scientific periodicals from various areas of health and from different parts of the world, our results were comprehensive, furnishing a general view of how the orientations to the authors for submitting a scientific article are made.

## OBJECTIVE

To analyze the ethical standards adopted by scientific journals in the healthcare area for publication of articles, and with this, prepare the Ethics Requirement Score, a bibliometric score to be applied to each scientific publication in the healthcare area in order to evaluate criteria applicable to ethics in scientific publication, in addition to evaluating the effects of the Impact Factor on the ethical requirements for publication in these journals.

## METHODS

This study considered as experimental units scientific publications in the healthcare area. These journals were selected by means of the databank of the Journal of Citation Reports^®^ (JCR) dated 2010 (available at: http://thomsonreuters.com/products_services/science/science_products/a/journal_citation_reports/). The JCR is a publication the evaluates the scientific impact of journals.^(26 )^ There was approval by the CEP of the *Universidade Federal de Ciências da Saúde de Porto Alegre* , registered under # 1582/12. An Informed Consent Form (ICF) for the conduction of this study was not necessary, since the data consulted were of public domain. All the journals were first analyzed as to inclusion and exclusion factors. The inclusion criterion was to be a scientific journal in the area of healthcare. The exclusion criteria were foreign scientific publications that did not have the same guidelines as the authors described in English, since it is the language adopted internationally, by the scientific community; scientific journals that had not published studies with human beings and did not have guidelines for authors available on the internet.

For the journals considered, data related to the IF and nationality (national or foreign) was obtained. First, all the journals of national origin with an IF duly calculated by the JCR were selected. For these, inclusion and exclusion criteria were applied to obtain the final list of Brazilian publications to be considered. Next, the same quantity of foreign origin journals were selected, considering the same criteria previously cited, ordered as per the IF.

Parameters related to ethics in publication were analyzed for each journal. For this, guides, norms, and instructions to the authors, available on the website of each publication, were analyzed. The parameters analyzed were approval by a CEP, Declaration of Helsinki and/or Resolution 196/96, recommendations as to plagiarism, need for application of the ICF, declaration of guarantee of confidentiality of the patients, registration in a database for clinical trials (when applicable), declaration of conflict of interest, and declaration of funding sources. Details as to each one of the items listed are described on chart 1 . Each item was analyzed considering its presence or absence. The date of each access at each website was recorded. The items on chart 1 were chosen based on a bibliographic review in articles with objectives similar to those of this article and served as inspiration. Namely, “Ethics in publication of research on human visceral leishmaniasis in national journals,”^(27 )^ “Ethical Standards adopted by Brazilian scientific journals,”^(23 )^ “Laying ethical foundations for clinical research,”^(28 )^ and “Brazilian journals that publish scientific articles on surgery. III: analysis of the instructions to authors based on the structure of requirements of Vancouver.”^(16 )^

**Chart 1:** Items evaluated for the preparation of the Ethics Requirement Score

Approval by CEP	Require approval by a CEP, as per the Declaration of Helsinki, published in 1964
Declaration of Helsinki and/or Resolution 196/96	Mention some of these documents that regulate ethics in research
Recommendations alerting about plagiarism	Contain an item recommended by the author as to plagiarism
ICF	Require that signing of an ICF be applied to all research subjects, as per the Declaration of Helsinki published in 1964
Confidentiality of patients and data	Contain an item recommending confidentiality about the data from the subjects involved in the research and their data
Recording in databases for clinical trials	Contain recommendations of a register of clinical trials in a database
Declaration of conflicts of interests	Contain orientation in reference to the interests of the said study
Declaration of funding	Contain orientation that makes clear the financial sources of the said study
ICMJE/COPE	Refer to the ICMJE or the COPE, which are related to ethics in scientific publications

CEP: Research Ethics Committee; ICF: Informed Consent Form; ICMJE: International Committee of Medical Journal Editors, COPE: Committee on Publication Ethics.

Based on all the ethical standards analyzed, except the item related to the Declaration of Helsinki and Resolution 196/96, the ERS was calculated. This score is given by the absolute quantity of items included among those considered for each journal. For this calculation, all the items had equivalent value, so that the ERS might represent values between zero and 8. Both the IF and the ERS were compared considering the fact of the journals being national or not as an independent variable. In this case, the periodicals were divided into two distinct groups formed by the national and foreign journals. In the same way, these variables were compared having as independent variable the character of the publications. In this case, the journals were stratified among those that published only reviews of theoretical articles and those that published results from original investigations. In both cases, Student’s t test was used for comparison among groups.

Additionally, the isolated frequency of each item among the national and foreign journals, and among the review periodicals and those dedicated to original data were analyzed by means of the χ^2^ test with Yates’s correction. Finally, the data related to the IF and ERS were correlated by means of Pearson’s correlation tests. In all cases, the statistically significant level adopted was p<0.05.

## RESULTS

Among the 89 Brazilian scientific publications with IF duly calculated by JCR, 52 were excluded from this study − 51 were excluded for being specific to other areas of research not related to health and one for not having instructions for the authors. In this way, at the end of the step, 37 national publications remained to be considered. These publications were paired with 37 foreign journals, ranked by IF (from the greatest to the smallest). In this way, the total sample was composed of the 37 best Brazilian periodicals of the healthcare area, ordered according the IF, and by the 37 best foreign journals of the healthcare area, also selected by IF, as is demonstrated on table 1 .

**Table 1:** Data relative to the impact factor and the Ethics Requirement Score

	Impact Factor			ERS	
	n	Mean	SD	Mean	SD
Foreign	37	29.84	13.69	3.97	2.05
National	37	0.76	0.42	3.32	1.78
Review	19	27.31	8.42	2.53	1.35
Original	54	11.32	18.09	4.09	1.93
Foreign and review	19	27.31	8.42	2.53	1.35
Foreign and original	18	32.51	17.03	5.50	1.42
National and review	0	0	0	0	0
National and original	37	1.28	0.59	3.32	1.76

Total	74				

SD: standart deviation; ERS: Ethics Requirement Score.

**Figure 1:**
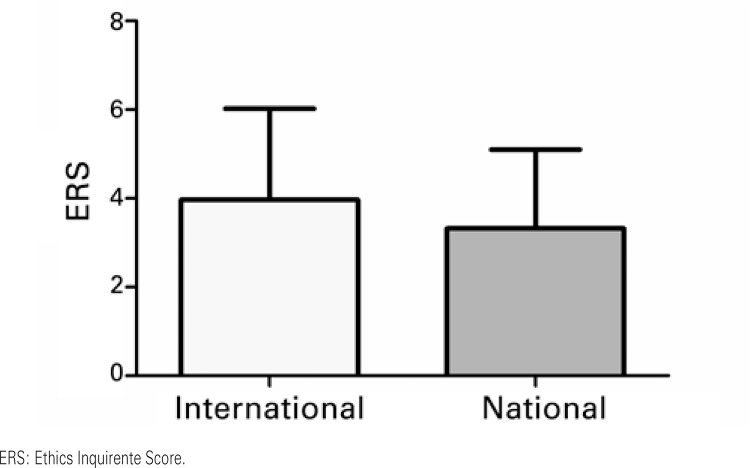
Effect of nationality of the journals as to the Ethics Requirement Score. Results based on Student’s t test. *p<0.05

**Figure 2:**
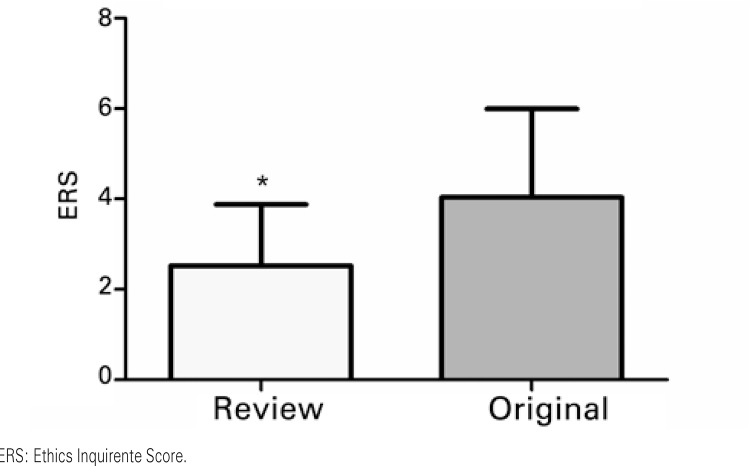
Effect of the type of journal on the Ethics Requirement Score. Results based on Student’s t test. * p<0.05

As to the comparisons, it was noted that the foreign publications had a significantly higher IF than the national journals, although no significant result was observed relative to the ERS ( Figure 1 ). As to the comparisons made when considering the dichotomy between the journals that published review articles and those that published results coming from original investigations, statistically significant effects were noted, both as to IF and as to ERS ( Figure 2 ).

As to the analysis of frequency of the ethical parameters considered, it was noted that national publications referred more frequently to documents such as the Declaration of Helsinki than organizations such as ICMJE and COPE, when compared to international publications. On the other hand, items related to plagiarism and the declaration of funding sources were more frequently encountered in foreign journals when compared to the national publications. As to review journals, these referred with a significantly lower frequency to publications of original investigations relative to the items on the Declaration of Helsinki, approval by CEP, policies on plagiarism, confidentiality of patients and data, indexation in clinical trial databases, and organizations such as ICMJE and COPE. The frequencies of each item and the result of the comparisons by means of the χ^2 ^test are presented on table 2 .

**Table 2:** Frequency of the ethical parameters analyzed and results obtained by means of the χ2 test

	Foreign	National	p value	Review	Original	p value
Declaration of Helsinki or Resolution 196/96	3	16	0.001	1	18	0.036
CEP	17	25	0.073	1	41	<0.001
Plagiarism	17	2	<0.001	9	10	0.031
ICF	19	12	0.187	1	30	<0.001
Confidentiality of the patients and data	5	2	0.449	1	6	0.771
Clinical study basis	12	12	1.000	1	23	0.007
Conflict of interests	34	26	0.059	17	43	0.538
Funding sources	36	25	0.004	18	43	0.243
ICMJE/COPE	7	18	0.011	0	25	<0.001

CEP: Committee of Ethics in Research; ICF: Informed Consent Form; ICMJE: International Committee of Medical Journals Editors; COPE: Committee on Publishing Ethics.

**Figure 3:**
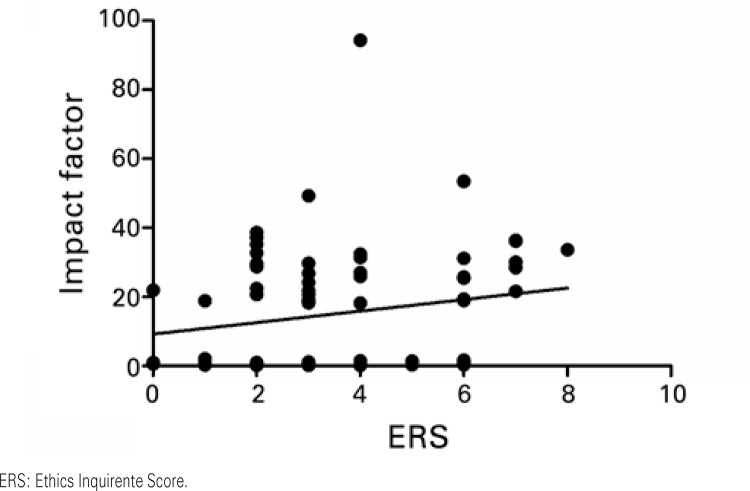
Correlations between the Impact Factor and the Ethics Requirement Score. Results based on Pearson’s correlation. r=0.184; p=0.116

**Figure 4:**
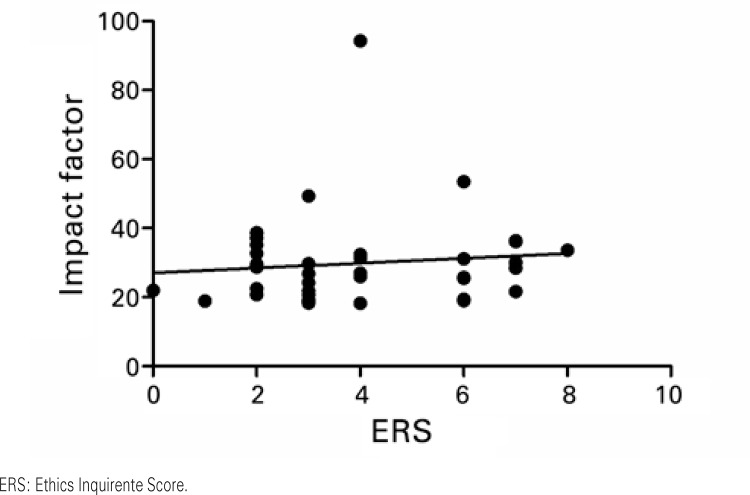
Correlation between the Impact Factor and the Ethics Requirement Score among the foreign publications. Results based on Pearson’s correlation. r=0.106; p=0.533

**Figure 5:**
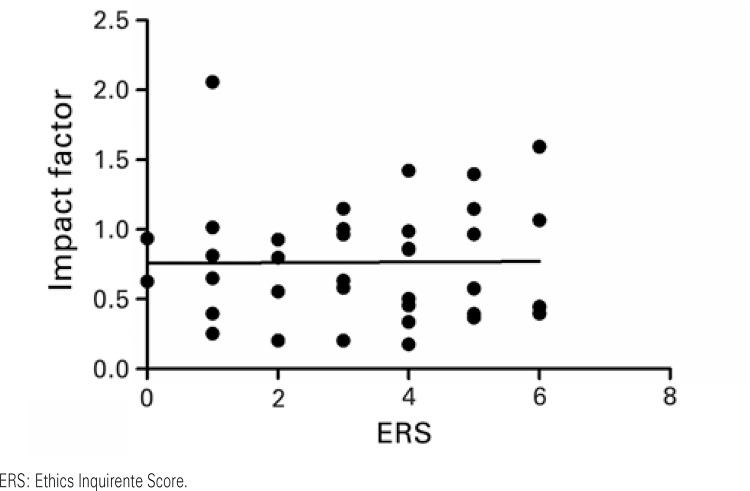
Correlation between the Impact Factor and the Ethics Requirement Score among the national journals. Results based on Pearson’s correlation. r=0.149; p=0.384

When IF and ERS were correlated, a weak correlation coefficient was observed with no statistical significance (r=0.184; p=0.116, as per Figure 3 ). Similar results were found in the analog correlations made specifically with the foreign (r=0.106; p=0.533, as per Figure 4 ) and national (r=0.149; p=0.384, as per Figure 5 ) publications. Pearson’s correlation test analyzes the correlation between the two numerical variables. It does not trace any cause and effect relation, but states only if they are “proportional”, both directly and indirectly. We obtained weak and non-significant correlations, showing that IF and ERS are not correlated, *i.e.* , there is no relationship of dependency between the values of IF and ERS in the journals analyzed.

## DISCUSSION

It is known that the great advancement in the biomedical area over the last few years has been accompanied by an intense discussion on the ethical conduction of the research carried out.^(29 )^ This project presented the effect of nationality (national or foreign) and type of publication (review articles or original articles) on the IF and on various parameters related to ethics in publication. Additionally, the proposal of ERS deserves to be underlined, a biometric index with the function of evaluating scientific periodicals as to the level of ethical requirements in the articles in which they are published.

All the items analyzed in this article are described in detail on chart 1 . For preparation of the ERS, all the items were considered, with exception of the item “Declaration of Helsinki/Resolution 196/96”. This item was not considered since it referred more to ethics in conducting the research than to ethics in publication. Additionally, the primary points covered by the Declaration of Helsinki and by Resolution 196/96 are treated by the other items computed by the creation of the ERS. Even so, the item “Declaration of Helsinki/Resolution 196/96” was observed in some periodicals, as is reported on table 2 , and is more frequent in national periodicals.

Even if the IF of foreign publications is considerably greater than that of the national publications, the results showed that the IF showed no significant correlation as to the score proposed. This fact allowed us to affirm that the ethics requirement for the publication is not related to the importance and the scope of the journal, in discordance with what was thought before the research was done. Since IF did not accompany the levels of ethical requirements, one can infer that the primary current bibliometric indexes still do not relate to the ethical requirements made by the periodicals. Thus, ERS evaluates a factor for which there still is no method of efficient measurement: the ethics requirement applied to publications in the area of healthcare.

All the national periodicals with IF refer to original investments which do not exclude them from publishing review articles, but they are not journals directed towards the publication of review articles. The review periodicals focus on some of the items proposed for the ERS, referring less to the Declaration of Helsinki, the approval from CEP, to the ICF, to the confidentiality of patients, and to the basis of clinical trials – which is reasonably comprehensible, considering that in reviews, there is no research that directly involves subjects or animals for there to be such concerns. However, only one foreign review journal, with an IF of 28.417, contemplated almost all the items proposed by the ERS, except the ICMJE/COPE. This allows us to think that, if a journal, even a review publication, has such ethical concerns, the others could and should have as well. Only one foreign journal and of original investigations, with an IF of 33.633, covered all the items. On the other hand, two national and one foreign journal did not cover any of the items proposed on chart 1 , whereas the three periodicals referred to original investigation, i.e., three periodicals did not even require that the research be approved by a CEP, which is unfortunate for science.

We perceived that various periodicals have submission stages in which the instructions were furnished and, in face of registration in the journal, the authors could continue in the process to submit or not. If there were details or more ethical instructions in the next steps, these were not recorded by this paper. Even if this fact is a limitation of this study, it is also a bias of the publication, since the norms should be exposed in the clearest way possible, so that the ethical requirements are clarified at the first contact with the author, that is, in the norms for publication.

## CONCLUSION

The progression of science is related to scientific research and consequently, to the publication of its results. However, the ethics criteria related to the conduction of research as well as interests and the information involved that accompany the dissemination of these results, should be equally required for publication. Even if the results showed that the Impact Factor did not interfere in the Ethics Requirement Score, it would be important to standardize the ethical orientations related to research with human beings, since this would stimulate the fulfillment of what has been established in documents that deal with human experimentation. However, in order to obtain the expected integrity in research and in publications, above all, an ethical posture is needed.
