# Supplementary material for: Ethics Requirement Score: new tool for evaluating ethics in publications
Source: Einstein (Sao Paulo). 2014 Oct-Dec;12(4):405–12. doi: 10.1590/S1679-45082014AO3001 (PMC4879904; doi:10.1590/S1679-45082014AO3001)
Supplement: Translation

# Translation: Escore de Exigência Ética: nova ferramenta para avaliação da ética em publicações

**Authors:** Lígia Gabrielle dos Santos, Ana Carolina da Costa e Fonseca, Claudia Giuliano Bica

**Affiliations:** 1 Universidade Federal de Ciências da Saúde de Porto Alegre, Porto Alegre, RS, Brasil.

**Keywords:** Ética, Ética em pesquisa, Fator de impacto, Publicações científicas e técnicas

## Abstract

**Objetivo:**

Analisar os padrões éticos adotados por periódicos científicos da área da saúde para publicação de artigos e elaborar o Escore de Exigência Ética, um índice bibliométrico a ser aplicado aos periódicos científicos da área da saúde visando avaliar critérios aplicáveis à ética na publicação científica.

**Métodos:**

Este trabalho considerou como unidades experimentais publicações científicas da área da saúde que foram selecionadas pelo banco de dados do *Journal of Citation Reports* ^®^2010. Parâmetros relacionados à ética na publicação foram analisados para cada revista. Para isso, foram analisados guias, normas e instruções aos autores, disponíveis no *website* de cada revista. Os parâmetros analisados foram: aprovação de um Comitê de Ética em Pesquisa, referência à Declaração de Helsinque e/ou à Resolução 196/96, recomendações sobre plágio, necessidade de aplicação do Termo de Consentimento Livre e Esclarecido, declaração de garantia de sigilo dos pacientes, registro em base de dados para ensaios clínicos (quando aplicável), declaração de conflito de interesses e declaração de fontes de financiamento. Cada item foi analisado considerando sua presença ou ausência.

**Resultados:**

Revistas estrangeiras possuíam Fator de Impacto significativamente maior que as revistas nacionais, contudo não foi observado resultado significativo em relação ao Escore de Exigência Ética.

**Conclusão:**

Ainda que o Fator de Impacto das revistas estrangeiras tenha sido consideravelmente maior do que o das nacionais, os resultados mostraram que o Fator de Impacto não apresentou resultado significativo em relação ao escore proposto, o que permite afirmar que a exigência ética para publicação não está relacionada com a abrangência e nem com alcance do periódico.

## INTRODUÇÃO

### Ética em pesquisa

É inegável que, nos últimos 100 anos, a ciência biomédica evoluiu substancialmente, devido ao entendimento de que a pesquisa é necessária, todavia, esse grande avanço depende da experimentação.^(1)^Ao conjunto de atividades que têm como objetivo desenvolver e contribuir para o conhecimento generalizável dá-se o nome de “pesquisa”. Conhecimento generalizável pode ser entendido como teorias, princípios ou relações, ou como o acúmulo de informações sobre as quais está baseado, as quais podem ser comprovadas por métodos científicos.^(2,3)^Entendendo a necessidade e a importância da experimentação na pesquisa, a grande questão é como conduzi-la de forma adequada e, em certo sentido, ética.

Ao longo da história, importantes escritos antigos (desde a época de Hipócrates), medievais e modernos demonstraram preocupação com cuidados em saúde, entretanto, somente pela metade do século XX, surgiu o primeiro documento de caráter internacional, com normas para a pesquisa médica com seres humanos:^(4)^o Código de Nuremberg.^(5)^Esse documento foi referendado por diversos documentos posteriores, como a Declaração de Helsinque (1964) em todas as suas versões, o Relatório Belmont (1978), as Diretrizes Internacionais para Pesquisa Biomédica em Seres Humanos (tradução brasileira, 1993) e, no Brasil, a Resolução do Conselho Nacional de Saúde (CNS) 196/96.^(6)^No ano de 1947, o Código de Nuremberg foi redigido por médicos estadunidenses cujo objetivo foi dar subsídios aos juízes do Tribunal de Nuremberg para o julgamento dos chamados crimes contra humanidade cometidos em pesquisas que foram conduzidas em campos de concentração,^(7 - 10)^tornando-se, assim, o primeiro código de conduta em pesquisas que viria a ser internacionalmente aceito.^(11)^

O Código de Nuremberg é composto por dez princípios que visam resguardar os direitos dos sujeitos que participam de pesquisas. O aspecto mais importante desse código é a obrigatoriedade do consentimento informado na pesquisa com seres humanos. Posteriormente, os princípios contidos no Código de Nuremberg passaram a integrar os códigos de ética dos profissionais que realizam pesquisa, tendo em vista sua regulamentação.^(12)^

Devido à necessidade de regulamentação das pesquisas com seres humanos, com vistas è proteção das populações a elas submetidas e devido à pouca influência do Código de Nuremberg sobre as práticas de pesquisa, surgiu a Declaração de Helsinque.^(2)^A Declaração de Helsinque foi publicada em 1964, durante a 18ª Assembleia Médica Mundial, realizada em Helsinque e, posteriormente, revisada em 1975 (Tóquio), 1983 (Veneza), 1989 (Hong Kong), 1996 (Sommerset West), 2000 (Edimburgo) e 2008 (Seul), e destaca a importância dos princípios éticos na condução de pesquisas com seres humanos, estabelecendo aspectos como a obtenção do Consentimento Informado e a apreciação do protocolo experimental por um comitê independente do investigador, conforme os itens 15 e 24 (World Medical Association: Declaration of Helsinki, 1964).^(13)^Afirma ainda que experimentos fora das normas dessa declaração não devem ser aceitos para publicação. Pesquisas com animais, nesse período, ainda parecem não ter problemas éticos.

Em 1978, foi divulgado o Relatório Belmont, que é considerado um marco teórico para a prática de pesquisa e que deu origem à teoria dos princípios, proposta por Tom Beauchamp e James Childress no livro *Princípios de ética biomédica* publicado em 1979. O Relatório Belmont apontou os seguintes princípios como referência fundamental para a ética na pesquisa:

– Respeito pelas pessoas, que encontra a sua correspondência prática na formulação e obtenção do Termo de Consentimento Informado;
– Beneficência, que pressupõe segurança e bem-estar aos participantes pela avaliação criteriosa da relação custo-benefício por sua inserção na pesquisa;
– Justiça no sentido liberal de equidade e traduzida pela possibilidade de igualdade de acesso à participação nos estudos e na distribuição dos resultados.^( ^7^ )^

Em 10 de outubro de 1996, foi publicada, no Brasil a Resolução CNS 196/96 que criou os Comitês de Ética em Pesquisa (CEPs), vinculados às instituições de pesquisas e exigindo que sua composição fosse multidisciplinar, incluindo, obrigatoriamente, um representante da comunidade. Essa resolução constituiu-se em um marco regulatório para as pesquisas científicas brasileiras envolvendo a experimentação humana. Tal resolução foi elaborada visando à proteção dos participantes dos estudos, à garantia dos direitos e dos deveres dos cientistas, de patrocinadores, das instituições envolvidas e do Estado. Foi criada também a Comissão Nacional de Ética em Pesquisa (CONEP), órgão máximo da área, ligado ao CNS – Ministério da Saúde (MS). É importante salientar o quão tardio foi esse processo no Brasil, uma vez que, antes desses órgãos e regulamentações, que datam de 1996, pesquisas já eram conduzidas. Recentemente, a Resolução 196/96 foi revogada pela resolução 466, de 12 de dezembro de 2012, e, apesar de apresentar alterações importantes, há problemas graves de redação. Essa nova versão não foi usada neste trabalho por ter sido publicada depois da coleta dos dados.

### Ética para publicação em periódicos científicos

A preocupação com a integridade e completude dos relatórios científicos é antiga. No quarto princípio metodológico estabelecido por Reneé Descartes, em seu *Discurso do método*, sugere que se deve “efetuar em toda parte relações metódicas tão completas e revisões tão gerais nas quais eu tivesse a certeza de nada omitir”.^(14)^Com base nesse princípio, nota-se a importância evidente da consistência e da integridade dos relatos científicos. Obviamente, a dinâmica das publicações científicas atuais difere da observada durante o século XV, quando, além da necessidade de relatos íntegros, outros itens tornaram-se importantes.

Atualmente, a credibilidade e o prestígio de um periódico científico estão relacionados ao rigor da política editorial, com a publicação de estudos que tenham sido conduzidos com rigor ético e científico, e que tenham potencial para influenciar no desenvolvimento da área de pesquisa em que se insere.^(15)^

As características estruturais para o envio de um artigo variam de acordo com o periódico, o que, atualmente, obriga os editores a estabelecerem instruções padronizadas, tentando uniformizar o formato de publicação. A dinâmica da ciência divulgada pelas revistas começou a ser objeto de análise de editores que sentiam necessidade de estruturar as publicações segundo algumas diretrizes que, posteriormente, transformaram-se em recomendações e normas.^(15)^Nesse contexto, deve-se destacar o trabalho realizado pelo *International Committee of Medical Journal Editors* (ICMJE), mais conhecido como Grupo de Vancouver, com a publicação dos *Uniform Requirements for Manuscripts Submitted to Biomedical Journals: Writing and Editing for Biomedical Journals*, que, desde 1978, procura estabelecer diretrizes para padronização e características das revistas biomédicas.^(15)^Entretanto, com o passar dos anos, apareceram outras preocupações que foram além do preparo de manuscritos. O intuito das edições mais recentes é esclarecer e direcionar interesses sobre direitos, privacidade, descrição de métodos, dentre outros assuntos.^(17)^

Mais recentemente, em 1997, foi criado o *Committee on Publication Ethics* (COPE) por um pequeno grupo de editores do Reino Unido. Atualmente com mais de 7.000 membros pelo mundo em diferentes áreas acadêmicas, o COPE oferece aconselhamento para editores e editoras sobre todos os aspectos éticos em publicações científicas e, em particular, sobre como lidar com casos de má conduta em pesquisa e publicação.^(18)^

Com o grande aumento na produção científica mundial, aumenta também a preocupação da comunidade científica com as transgressões éticas nas publicações científicas. Devido ao fato de a pesquisa estar vinculada a valores e normas, espera-se que o pesquisador a conduza de forma íntegra e que garanta padrões científicos de excelência e de confiança em seu desenvolvimento.^(19)^As violações graves desse comportamento passaram a ser conhecidas como “má conduta científica”, e podem ser resumidas em fabricação, falsificação ou plágio na elaboração de proposta, na realização ou na avaliação de pesquisa ou, ainda, no relato de resultados de pesquisa.^(18)^

Devido ao grande número de ensaios clínicos, ao investimento das indústrias farmacêuticas na pesquisa de novos medicamentos e aos possíveis problemas éticos aplicáveis a esses casos (como a omissão e não publicação de dados) o *Consolidated Standards of Reporting Trials* (CONSORT) foi criado pelo *Consort Group* . O CONSORT elenca e sistematiza diversas iniciativas desenvolvidas para solucionar problemas decorrentes da forma inadequada de fornecimento de dados em ensaios clínicos randomizados.^(20)^Dessa forma, para transparência de um ensaio clínico, ele deve ser registrado em uma base de dados que siga as diretrizes do CONSORT, como, por exemplo, o *Australian Clinical Trials Registry* , o *Clinical Trials* , o *Nederlands Trial Register* e *UMIN Clinical Trials Registry* , que são as maiores bases de dados mundiais. A cada ensaio clínico randomizado indexado a uma dessas bases de dados, confere-se um número de registro. Esse registro confere confiabilidade aos resultados, uma vez que, independente do desfecho do estudo, seus resultados são registrados. Em nenhum periódico foi citado o registro brasileiro de ensaios clínicos, o ReBEC.

O aumento da pressão sobre os pesquisadores para que publiquem a qualquer custo, tendo como objetivos o prestígio e mais verba para pesquisa, contribuiu para grande parte do crescimento da fraude na pesquisa.^(19)^Um estudo do provedor de dados acadêmicos Thomson Reuters mostrou que o número de artigos publicados em periódicos com sistema de avaliação por pares nos últimos 20 anos dobrou, enquanto o número de retratações aumentou 20 vezes no mesmo período, possivelmente como consequência do aparecimento de melhores sistemas de detecção de plágio como o *Déjà vu* , Ephorus, Jplag e Etblast, por exemplo,^(21)^e também pela cobrança que os editores têm recebido para medidas contra má conduta.^(22)^

No Brasil, não acontece de modo diferente. Devido ao aumento da produção científica, é cada vez maior a procura por publicações, preferencialmente em revistas com maior Fator de Impacto (FI).^(23)^Nesse sentido, diversos índices bibliométricos foram criados para quantificar e qualificar a produção científica, sendo aplicados a um pesquisador (considerando todos os seus artigos), a um artigo de modo independente, ou a uma publicação. Dentre esses índices, destacam-se o Índice H, o *Eigenfactor* e o Índice de Imediaticidade. Apesar da importância crescente desses índices à bibliometria e à cienciometria mundial, o índice mais utilizado é o FI. Esse índice, criado pelo *Insititute for Scientific Information* (ISI), parte da Thomson Reuters, é aplicado a cada publicação periódica e é basicamente uma razão entre citações recebidas e artigos publicados.^(24)^De modo detalhado, o FI é calculado pelo número de citações (C) que um periódico recebe em um determinado ano, dividido pelo número de todos os artigos neles publicados durante os dois anos posteriores (A). Desse modo, sua fórmula é: FI= C/A (http://thomsonreuters.com/products_ services/science/academic/impact_factor/).

Estudos que avaliam a produção científica possibilitam um panorama das políticas de gestão científica, facilitando, dessa forma, a compreensão da disseminação do conhecimento científico.^(25)^Dentro desse contexto de aumento da produção científica mundial e em face dos casos de fraude em publicação, é necessário que seja valorizada a exigência ética como uma característica que agregue qualidade às publicações científicas.

Este trabalho se propôs a criar um Escore de Exigência Ética (EEE) com o intuito de ser um índice que avalie a exigência ética dos periódicos científicos da área da saúde. Mais importante do que publicar é a preocupação com o respeito e com a integridade na forma como a pesquisa é conduzida. O EEE nos deve mostrar se existe associação entre o FI do periódico e as exigências éticas nas instruções fornecidas aos autores.

A relevância deste trabalho esteve em verificar se os aspectos éticos que visam a benefícios e aos direitos dos sujeitos de pesquisa são respeitados, e se os interesses de patrocinadores, pesquisadores e investigadores são devidamente relatados, dentre outros aspectos. Devido à consulta de periódicos científicos de diversas áreas da saúde e de diversos lugares do mundo, nossos resultados foram abrangentes fornecendo uma visão geral de como são feitas as orientações aos autores para submissão de um artigo científico.

## OBJETIVO

Analisar os padrões éticos adotados por periódicos científicos da área da saúde para publicação de artigos e, com isso, elaborar o Escore de Exigência Ética, um índice bibliométrico a ser aplicado a cada periódico científico da área da saúde para avaliar critérios aplicáveis à ética na publicação científica, além de avaliar o efeito do Fator de Impacto sobre as exigências éticas para publicação nesses periódicos.

## MÉTODOS

Este trabalho considerou como unidades experimentais publicações científicas da área da saúde. Esses periódicos foram selecionados por meio do banco de dados do *Journal of Citation Reports*^®^(JCR) de 2010 (disponível em: http://thomsonreuters.com/products_services/science/science_products/a/journal_citation_reports/). O JCR é uma publicação que avalia o impacto científico de periódicos.^(26)^ Houve aprovação pelo CEP da Universidade Federal de Ciências da Saúde de Porto Alegre, com registro sob parecer 1582/12. Um Termo de Consentimento Livre e Esclarecido (TCLE) para realização deste trabalho foi desnecessário, visto que os dados consultados eram de domínio público. Todas as revistas foram primeiramente analisadas em relação aos fatores de inclusão e de exclusão. O critério de inclusão foi ser periódico científico da área da saúde. Já os critérios de exclusão foram ser periódico científico estrangeiro que não tivesse as orientações aos autores descritas em inglês, por ser a língua adotada internacionalmente pela comunidade científica; ser periódico científico que não publicasse estudos com seres humanos; não possuir orientações aos autores disponíveis na internet.

Para as revistas consideradas, foram obtidos dados relacionados ao FI e à nacionalidade (nacional ou estrangeira). Primeiramente, foram selecionadas todas as revistas de origem nacional com FI devidamente calculado pelo JCR. Para estas, foram aplicados os critérios de inclusão e de exclusão para obtenção da lista final de publicações brasileiras a serem consideradas. Depois disso, foi selecionada a mesma quantidade de revistas de origem estrangeira, considerando os mesmos critérios previamente citados, ordenadas de acordo com o FI.

Parâmetros relacionados à ética na publicação foram analisados para cada revista. Para isso, foram analisados guias, normas e instruções aos autores, disponíveis no *website* de cada revista. Os parâmetros analisados foram: aprovação por um CEP, Declaração de Helsinque e/ou Resolução 196/96, recomendações sobre plágio, necessidade de aplicação do TCLE, declaração de garantia de sigilo dos pacientes; registro em base de dados para ensaios clínicos (quando aplicável), declaração de conflito de interesses e declaração de fontes de financiamento. Detalhes sobre cada um dos itens relacionados são descritos no quadro 1. Cada item foi analisado considerando sua presença ou ausência. A data de cada acesso em cada *website* foi registrada. Os itens do quadro 1foram escolhidos com base em uma revisão bibliográfica em artigos que tinham objetivos parecidos com os deste trabalho e que serviram como inspiração. Foram eles: “Ética na publicação de pesquisas sobre leishmaniose visceral humana em periódicos nacionais”,^(27)^“Padrões Éticos adotados pelas revistas científicas brasileiras”,^(23)^“ *Laying ethical foundations for clinical research* ”,^(28)^e “Revistas brasileiras publicadoras de artigos científicos em cirurgia. III: análise das instruções aos autores baseada na estrutura dos requisitos de Vancouver”.^(16)^

**Quadro 1:** Itens avaliados para a elaboração do Escore de Exigência Ética

Aprovação do CEP	Exigir aprovação por um CEP, conforme a Declaração de Helsinque, publicada em 1964
Declaração de Helsinque e/ou Resolução 196/96	Mencionar alguns desses documentos que regulamentam a ética em pesquisa
Recomendações alertando sobre plágio	Conter algum item alertando o autor sobre plágio
TCLE	Exigir que tivesse sido aplicado aos sujeitos de pesquisa um TCLE, conforme a Declaração de Helsinque publicada em 1964
Sigilo de pacientes e dados	Conter um item recomendando o sigilo dos dados dos sujeitos envolvidos na pesquisa e seus dados
Registro em base de dados para ensaios clínicos	Conter recomendação de registro de ensaios clínicos em uma base de dados
Declaração de conflito de interesses	Conter uma orientação referindo-se aos interesses do estudo em questão
Declaração de financiamento	Conter uma orientação que deixe claro as fontes financiadoras do estudo em questão
ICMJE/COPE	Referir-se ao ICMJE ou ao COPE, que estão relacionados à ética em publicações científicas
CEP: Comitê de Ética em Pesquisa; TCLE: Termo de Consentimento Livre e Esclarecido; ICMJE: *International Committee* *of Medical Journal Editors* , COPE: *Committee on Publication Ethics* .	

Com base em todos os padrões éticos analisados, exceto o item relacionado à Declaração de Helsinque e à Resolução 196/96, foi calculado o EEE. Esse escore é dado pela quantidade absoluta de itens contemplados dentre os considerados para cada revista. Para esse cálculo, todos os itens obtiveram valor equivalente, de modo que o EEE pudesse apresentar valores entre zero a 8. Tanto o FI quanto o EEE foram comparados tendo o fato de as revistas serem nacionais ou não como variável independente. Nesse caso, as revistas foram distribuídas em dois grupos distintos compostos pelas revistas nacionais e pelas estrangeiras. Da mesma forma, essas variáveis foram comparadas tendo como variável independente o caráter das publicações. Nesse caso, as revistas foram estratificadas entre aquelas que publicam apenas revisões ou artigos teóricos e aquelas que publicam resultados advindos de investigações originais. Em ambos os casos empregou-se o teste *t de* Student para a comparação entre os grupos.

Além disso, a frequência isolada de cada item entre as revistas nacionais e estrangeiras, e entre as revistas de revisão e as dedicadas a dados originais foi analisada por meio do teste χ^2^com correção de Yates. Por fim, os dados relacionados ao FI e ao EEE foram correlacionados por meio de testes de correlações de Pearson. Em todos os casos, adotou-se como estatisticamente significativo p<0,05.

## RESULTADOS

Dentre as 89 revistas científicas brasileiras com FI devidamente calculado pelo JCR, 52 foram excluídas deste estudo − 51 foram excluídas por serem específicas a outras áreas de pesquisa não relacionadas às da saúde e 1 por não ter instruções para os autores. Desse modo, ao final da etapa da seleção restaram 37 revistas nacionais a serem consideradas. Essas revistas foram pareadas a 37 revistas estrangeiras, ranqueadas por FI (do maior para o menor). Desse modo, a amostra total foi composta pelas 37 melhores revistas brasileiras da área da saúde, ordenadas por FI, e pelas 37 melhores revistas estrangeiras da área da saúde, também selecionadas pelo FI, conforme demonstrado na tabela 1.

**Tabela 1:** Dados relativos ao fator de impacto e ao Escore de Exigência Ética

	Fator de impacto			EEE	
	n	Média	DP	Média	DP
Estrangeiras	37	29,84	13,69	3,97	2,05
Nacionais	37	0,76	0,42	3,32	1,78
Revisão	19	27,31	8,42	2,53	1,35
Originais	54	11,32	18,09	4,09	1,93
Estrangeiras e de revisão	19	27,31	8,42	2,53	1,35
Estrangeiras e originais	18	32,51	17,03	5,50	1,42
Nacionais e de revisão	0	0	0	0	0
Nacionais e originais	37	1,28	0,59	3,32	1,76
Total	74				
SD: desvio padrão; EEE: Escore de Exigência Ética.					

Quanto às comparações, observou-se que as revistas estrangeiras possuíam FI significativamente maior que as revistas nacionais, contudo não foi observado resultado significativo em relação ao EEE ( Figura 1). Quanto às comparações feitas quando considerada a dicotomização entre revistas que publicavam artigos de revisão e artigos que publicavam resultados advindos de investigações originais, observaram-se efeitos estatisticamente significativos, tanto em relação ao FI quanto ao EEE ( Figura 2).

**Figura 1:** Efeito da nacionalidade das revistas sobre o Escore de Exigência Ética. Resultados com base no teste *t* de Student. *p<0,05

**Figura 2:** Efeito do tipo de revista sobre o Escore de Exigência Ética. Resultados com base no teste *t* de Student. * p<0,05

Quanto à análise da frequência dos parâmetros éticos considerados, notou-se que as revistas nacionais referiram-se mais frequentemente a documentos como a Declaração de Helsinque e a organizações, como o ICMJE e o COPE, quando comparadas às revistas internacionais. Em contrapartida, itens relacionados ao plágio e à declaração de fontes de financiamento foram mais frequentemente encontrados nas revistas estrangeiras em comparação às nacionais. Quanto às revistas de revisão, estas se referiram com frequência significativamente menor às revistas de investigações originais, em relação aos itens sobre Declaração de Helsinque, aprovação por CEP, política de plágio, sigilo de pacientes e dados, indexação em base de ensaios clínicos e organização como ICMJE e COPE. As frequências de cada item e o resultado das comparações por meio do teste χ^2^são apresentadas na tabela 2.

**Tabela 2:** Frequência dos parâmetros éticos analisados e resultados obtidos por meio do teste χ2

	Estrangeiras	Nacionais	Valor de p	Revisão	Originais	Valor de p
Declaração de Helsinque ou Resolução 196/96	3	16	0,001	1	18	0,036
CEP	17	25	0,073	1	41	<0,001
Plágio	17	2	<0,001	9	10	0,031
TCLE	19	12	0,187	1	30	<0,001
Sigilo de pacientes e dados	5	2	0,449	1	6	0,771
Base de ensaios clínicos	12	12	1,000	1	23	0,007
Conflito de interesses	34	26	0,059	17	43	0,538
Fontes de financiamento	36	25	0,004	18	43	0,243
ICMJE/COPE	7	18	0,011	0	25	<0,001

CEP: Comitê de Ética em Pesquisa; TCLE: Termo de Consentimento Livre Esclarecido; ICMJE: *International Committee of Medical Journals Editors* ; COPE: *Committee on Publishing Ethics.*

Quando correlacionados FI e EEE, observou-se um coeficiente de correlação fraco e sem significância estatística (r=0,184; p=0,116, conforme Figura 3). Resultados semelhantes foram encontrados nas correlações análogas feitas especificamente com as revistas estrangeiras (r=0,106; p=0,533, conforme Figura 4) e nacionais (r=0,149; p=0,384, conforme Figura 5). O teste de correlação de Pearson analisa a correlação entre duas variáveis numéricas. Ele não traça nenhuma relação de causa ou efeito, apenas diz se são “proporcionais”, tanto direta quanto indiretamente. Obtivemos correlações fracas e não significativas, mostrando que FI e EEE não são correlacionados, ou seja, não há qualquer relação de dependência entre os valores de FI e EEE nas revistas analisadas.

**Figura 3:** Correlação entre Fator de Impacto e Escore de Exigência Ética. Resultados com base em correlação de Pearson. r=0,184; p=0,116

**Figura 4:** Correlação entre Fator de Impacto e Escore de Exigência Ética entre as revistas estrangeiras. Resultados com base em correlação de Pearson. r=0,106; p=0,533

**Figura 5:** Correlação entre Fator de Impacto e Escore de Exigência Ética entre as revistas nacionais. Resultados com base em correlação de Pearson. r=0,149; p=0,384

## DISCUSSÃO

Sabe-se que o grande avanço na área biomédica nos últimos anos tem sido acompanhado de intensa discussão sobre a condução ética das pesquisas realizadas.^(29)^Este trabalho apresentou os efeitos da nacionalidade (nacional ou estrangeira) e do tipo de publicação (artigos de revisão ou artigos originais) sobre o FI e sobre diversos parâmetros relacionados à ética em publicação. Além disso, merece destaque a proposta do EEE, um índice bibliométrico cuja função deve ser a de avaliar os periódicos científicos quanto ao nível de exigência ética requerido nos artigos neles publicados.

Todos os itens analisados neste trabalho são detalhadamente descritos no quadro 1. Para elaboração do EEE, todos os itens foram considerados, com exceção do item “Declaração de Helsinque/Resolução 196/96”. Esse item foi desconsiderado por se referir mais à ética em condução de pesquisa do que à ética em publicação. Além disso, os principais pontos abordados pela Declaração de Helsinque e pela Resolução 196/96 são tratados pelos outros itens computados para criação do EEE. Ainda assim, o item “Declaração de Helsinque/Resolução 196/96” foi observado em algumas revistas, conforme relatado na tabela 2, sendo mais frequente nos periódicos nacionais.

Ainda que o FI das revistas estrangeiras tenha sido consideravelmente maior do que o das nacionais, os resultados mostraram que o FI não apresentou correlação significativa no que concerne ao escore proposto. Esse dado nos permitiu afirmar que a exigência ética para publicação não está relacionada com a importância e com o alcance do periódico, contrariando o que se pensava antes da realização da pesquisa. Visto que o FI não acompanhou os níveis de exigência ética, pode-se inferir que os principais índices bibliométricos atuais não se relacionam à exigência ética feita pelos periódicos. Assim, o EEE avalia um fator para o qual ainda não se tem nenhum modo de medida eficiente: a exigência ética aplicada às publicações na área da saúde.

Todos os periódicos nacionais com FI referiram-se a investigações originais, o que não os excluem de publicar artigos de revisão, entretanto, não são revistas direcionadas à publicação de artigos de revisão. Os periódicos de revisão contemplam alguns dos itens propostos para o EEE, referindo-se menos à Declaração de Helsinque, à aprovação de CEP, ao TCLE, ao sigilo dos pacientes e à base de ensaios clínicos − o que é razoavelmente compreensível, visto que, em revisões, não há uma pesquisa que envolva diretamente sujeitos ou animais para que existissem tais preocupações. Entretanto, apenas um periódico estrangeiro de revisão, com FI 28.417 contemplou quase todos os itens propostos pelo EEE, exceto o ICMJE/COPE. Isso nos permite pensar que, se um periódico, mesmo sendo de revisão, tem tais preocupações éticas, os demais também poderiam e deveriam ter. Somente um periódico estrangeiro e de investigações originais, cujo FI era de 33.633, teve todos os itens contemplados. Em contrapartida, dois periódicos nacionais e um estrangeiro não contemplaram nenhum item proposto no quadro 1 , sendo que os três periódicos referem-se a investigações originais, ou seja, três periódicos sequer exigiam que a pesquisa apresentada tivesse sido aprovada por um CEP, o que é lastimável para a ciência.

Percebemos que diversos periódicos possuíam estágios de submissão em que as instruções eram fornecidas e, mediante um cadastro na revista, os autores podiam dar andamento ao processo de submissão. Caso existisse detalhamento ou mais instruções de caráter ético nos passos seguintes, estes não foram registrados por este trabalho. Ainda que esse fato seja uma limitação do estudo, também é um viés da revista, visto que as normas devem ser expostas do modo mais claro possível, para que as exigências éticas sejam esclarecidas no primeiro contato com o autor, ou seja, nas normas para publicação.

## CONCLUSÃO

A evolução da ciência está relacionada à pesquisa científica e, consequentemente à publicação de seus resultados. Entretanto, os critérios éticos relacionados à condução da pesquisa bem como interesses e as informações envolvidas, que acompanham a divulgação desses resultados, devem ser igualmente exigidos para publicação. Mesmo que os resultados tenham mostrado que o Fator de Impacto não interferiu no Escore de Exigência Ética, seria importante a padronização das orientações éticas relacionadas a pesquisas com seres humanos, pois isso estimularia o cumprimento do estabelecido em documentos que tratam a experimentação humana. Contudo, para obtenção da esperada integridade em pesquisa e em publicações, é necessária, acima de tudo, uma postura ética.
